# Musculoskeletal Growth Modulation in Gilthead Sea Bream Juveniles Reared at High Water Temperature and Fed with Palm and Rapeseed Oils-Based Diets

**DOI:** 10.3390/ani11020260

**Published:** 2021-01-21

**Authors:** Sara Balbuena-Pecino, Natàlia Riera-Heredia, Esther Gasch-Navalón, Albert Sánchez-Moya, Ramon Fontanillas, Joaquim Gutiérrez, Isabel Navarro, Encarnación Capilla

**Affiliations:** 1 Departament de Biologia Cellular, Fisiologia i Immunologia, Facultat de Biologia, Universitat de Barcelona, 08028 Barcelona, Spain; sara.balbuena@ub.edu (S.B.-P.); natalia.riera@gmail.com (N.R.-H.); esthergasch19@gmail.com (E.G.-N.); alsanchezmo@ub.edu (A.S.-M.); jgutierrez@ub.edu (J.G.); mnavarro@ub.edu (I.N.); 2 Skretting Aquaculture Research Centre, 4016 Stavanger, Norway; ramonfontanillas@gmail.com

**Keywords:** white muscle, bone, GH-IGFs system, vegetable oils, aquaculture, climate change

## Abstract

**Simple Summary:**

Diet optimization and global warming are two important challenges concerning fish farming. In the current study, both diets containing different vegetable oils (palm or rapeseed) and a situation of elevated water temperature (28 versus 21 °C) are evaluated in gilthead sea bream. The temperature increase caused a transcriptional modulation of development-related genes, while the palm and rapeseed oils-based diet appeared to be the most beneficial one, overall promoting an optimum endocrine environment for balanced musculoskeletal growth. Thus, data reveal the importance of considering diet formulation in a future climate change context to ensure sustainable production and welfare of aquatic animals.

**Abstract:**

The upward trend of seawater temperature has encouraged improving the knowledge of its consequences on fish, considering also the development of diets including vegetable ingredients as an approach to achieve a more sustainable aquaculture. This study aims to determine the effects on musculoskeletal growth of: (1) a high-water temperature of 28 °C (versus 21 °C) in gilthead sea bream juveniles (*Sparus aurata*) fed with a diet rich in palm oil and, (2) feeding the fish reared at 28 °C with two other diets containing rapeseed oil or an equilibrated combination of both vegetable oils. Somatic parameters and mRNA levels of growth hormone-insulin-like growth factors (GH-IGFs) axis-, osteogenic-, myogenic-, lipid metabolism- and oxidative stress-related genes in vertebra bone and/or white muscle are analyzed. Overall, the data indicate that high-water rearing temperature in this species leads to different adjustments through modulating the gene expression of members of the GH-IGFs axis (down-regulating *igf-1*, its receptors, and binding proteins) and also, to bone turnover (reducing the resorption-activity genes cathepsin K (*ctsk*) and matrix metalloproteinase-9 (*mmp9*)) to achieve harmonic musculoskeletal growth. Moreover, the combination of palm and rapeseed oils seems to be the most beneficial at high-water rearing temperature for both balanced somatic growth and muscular fatty acid uptake and oxidation.

## 1. Introduction

Aquaculture has been challenged to meet the increasing demand of global fish consumption, due to the growing human population. However, over the last few years, approximately 20 million tons of the world’s capture fisheries have been required for non-human food production, including transformation into fishmeal (FM) and fish oil (FO) for use in animal husbandry and fish aquaculture feeds. Consequently, the utilization of such a volume of raw material for this purpose rather than for direct human consumption has been under continued controversy and debate, emerging the need to explore alternative nutrient sources [1,2]. Therefore, reducing aquafeeds’ dependence on FM and FO is nowadays recognized as a priority for the future growth and sustainability of the industry [3].

FO is characterized by its high digestibility and content of n-3 long chain polyunsaturated fatty acids (LC-PUFA), such as eicosapentaenoic (EPA, 20:5n-3) and docosahexaenoic (DHA, 22:6n-3) acids. These are well-known to be essential for the optimal growth and health of marine species, since these fish have a low or absent capability to elongate and desaturate the two PUFA linoleic (LA, 18:2n-6) and α-linolenic (ALA, 18:3n-3) acids to the biologically active and essential LC-PUFA [3,4,5]. Currently, FO replacement by vegetable oils (VO) has come to the forefront, as they are considered promising candidates given their competitive price and ready availability. Research on FO substitution has indicated that it can be replaced by a range of several plant seed oils, among which, palm (PO) and rapeseed (RO, also referred to as canola) oils are, respectively, the first and the third most produced VO in the world [5,6]. However, at the same time, a great controversy exists regarding the production of PO, since due to its high and increasing global demand, the cultivation of this VO has led to multiple undesirable environmental effects such as deforestation, habitats and biodiversity losses, or pollution increase. This negative impact has called for the sustainable production of PO, and consequently, a growing number of certified concessions have been developed in the last years [7]. In addition, fine-tuning VO diet design for aquaculture is mandatory to assure coverage of the essential fatty acid (EFA) requirements in fish, which differ between species [1]. The most appropriate VO should contain high levels of saturated fatty acids (SFA) and monounsaturated fatty acids (MUFA) as energy sources, and low amounts of LA, since this fatty acid is poorly oxidized and difficult to use for energetic purposes [5]. Thus, PO and RO are considered good alternative dietary lipid sources for salmonids [8] and other freshwater and marine fish species [9,10,11], although there is no clear consensus on their effects among species. PO has relatively low levels of LA but high levels of palmitic (16:0) and oleic (18:1n-9) acids, whereas RO, although devoid of n-3 PUFA and relatively high in LA, is at the same time as rich in MUFA as oleic acid [10,12,13]. Besides, the proportion of n-3 LC-PUFA can be balanced in the diet with the inclusion of linseed oil (LO), since this oil contains more than 40% of n-3 fatty acid series. In this sense, our group recently found that blends of different VO (i.e., PO, RO, LO and soybean oil, SO) can be used in diets with up to 75% FO substitution with optimal results in gilthead sea bream (*Sparus aurata*) juveniles [14]; particularly, we point out that PO could be a promising VO, given its positive effect on growth while maintaining healthy metabolic conditions in this species.

Up to now, VO inclusion in partially or totally substituted FO diets has been evaluated in many fish species [15,16,17,18], including gilthead sea bream [10,14,19,20,21,22]. Although there is a large amount of literature about FO substitution in fish feed formulation, its impact in bone tissue growth has hardly been studied. Furthermore, as reported by the Intergovernmental Panel on Climate Change (IPCC), Earth’s climate system is warming, and the global mean sea surface temperature will increase more than 2 °C by the end of this century [23]. This is likely to significantly affect marine species in several ways, and very little is known about the effect of replacement diets within a scenario of increased water temperature. Recently, Pettersson et al. [24] investigated such interaction in a cold-water species, the Arctic charr (*Salvelinus alpinus*). Partial replacement of FO with a blend of RO and PO had no effect on growth but increased muscle total lipid content while reducing the swimming capacity of the fish, with major differences at low water temperature.

In this context, the objectives of the present research are to evaluate the effects on musculoskeletal growth in gilthead sea bream juveniles of: (1) an increased water temperature of 28 °C (versus 21 °C) in fish fed with a diet rich in PO and, (2) feeding the fish reared at high temperature (28 °C), with two other replacement FO diets containing RO or a combination of both VO, in addition to the PO-rich diet. The mRNA levels of the growth hormone-insulin-like growth factors (GH-IGFs) axis-, osteogenic-, myogenic-, lipid metabolism- and oxidative stress-related genes in bone and/or white muscle are determined. This study is performed to extend the knowledge about the importance of feed formulation in aquaculture in parallel to the possible impact of global climate change on musculoskeletal development in this species. In fact, this study is an extension of a previous work, in which somatic parameters, liver and adipose tissue cellularity and lipid metabolism-related genes expression, were characterized in the exact same experimental gilthead sea bream juveniles [22]. In that study, it was reported that the combination of fatty acids from PO and RO in the diet seemed to be the most equilibrated, since animals presented proper growth and balanced hepatic and adipose tissue lipid accumulation even at the elevated rearing temperature.

## 2. Materials and Methods

### 2.1. Animals, Experimental Diets and Ethics Statement

Gilthead sea bream (*S. aurata*) juveniles were obtained from the commercial hatchery Piscimar (Burriana, Spain) and maintained in the animal facilities (Spanish Operational Code REGA ES080/90036535) of the Faculty of Biology at the University of Barcelona (Barcelona, Spain). During the acclimation period (one month), fish were randomly distributed into 200 or 400 L tanks in a seawater recirculation system under a 12 h light/12 h dark photoperiod, 38‰ of salinity, in a 21 or 28 °C-controlled temperature room. Fish were fed ad libitum three times a day with a commercial diet (Optibream Skretting, Burgos, Spain).

**Figure 1:**
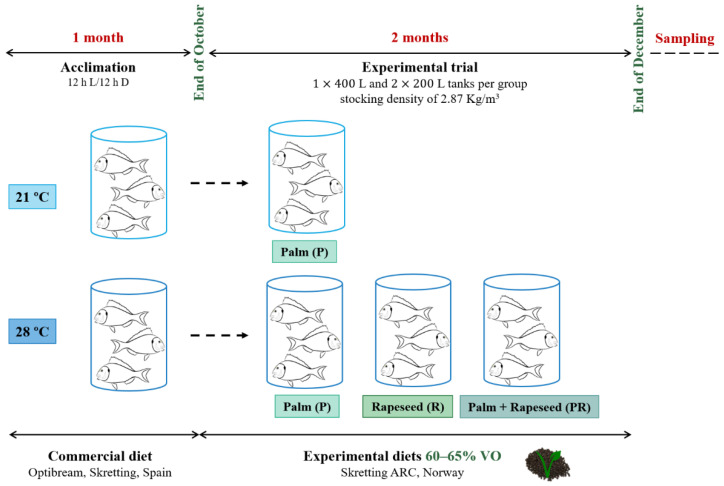
Schematic design of the experimental trial. Gilthead sea bream juveniles were held one month either at 21 or 28 °C in two different controlled temperature rooms for acclimation before feeding them with partially substituted FO diets (with 60% to 65% VO content of the total oils). From the end of October to the end of December, two groups of fish received a diet containing palm oil (P) and were maintained at 21 or 28 °C; while the remaining groups at 28 °C were fed either with a diet containing rapeseed oil (R) or a combination of both oils (PR). FO: fish oil; VO: vegetable oil.

After acclimation, a two-months trial (from the end of October to the end of December) was performed, as previously described [22]. The initial body weight and stocking density of the fish were 23.03 ± 0.41 g and 2.87 kg/m^3^, respectively. For the trial, three different partially substituted FO diets by VO were formulated and produced by Skretting ARC (Stavanger, Norway) (Table 1), with a 60–65% of the total oils being VO. The inclusion of a minimum of a 4.45% of FO, in addition to FM, ensured the minimum requirements of EPA and DHA for fish [25]; but in addition, all the diets were formulated with a similar content of LO (10–15% of the total oils) in order to balance the content in n-3 fatty acids. Two groups of fish were fed with a diet containing PO (named P) and held either at 21 or 28 °C in temperature controlled rooms, and the other two groups were maintained at 28 °C and were fed with diets including RO (R) or a combination of PO and RO (PR). Each experimental group was represented by triplicate tanks (one of 400 L, and two of 200 L, holding 55 and 27 fish each, respectively). Fish were fed thrice a day by automatic feeders with a daily rate of 2.5% body weight that was adjusted each week. A schema of the experimental trial is shown in Figure 1.

At the end of the trial, fish were fasted for 24 h prior sampling to deplete the gastrointestinal tract and avoid contamination of the tissues. All animals were anesthetized with MS-222 (150 mg/L) (E10521, Sigma-Aldrich, Tres-Cantos, Spain), measured and weighed to determine the biometric parameters. Then, 12 fish per condition (four from each tank) were sacrificed by cranial concussion and pieces of bone from the vertebral column and white muscle from the dorsal epaxial area were obtained and directly frozen in liquid nitrogen. All samples were stored at −80 °C until further analysis. All animal handling procedures carried out in this study complied with the Guidelines of the European Union Council directive (EU 2010/63) and were approved by the Ethics and Animal Care Committee of the University of Barcelona (permit numbers CEEA 110/17 and DAAM 9488), following the regulations and procedures established by the Spanish and Catalan governments.

### 2.2. Biometric Parameters

The different somatic parameters evaluated were determined as in Riera-Heredia et al. [22] using the following formulas: Initial body weight (IBW); final body weight (FBW); weight gain (WG) [(FBW − IBW/IBW) × 100]; somatic growth rate (SGR) [((ln FBW − ln IBW/time) × 100] where time was 53 days; body length (BL); condition factor (CF) [FBW/BL^3^ × 100]. Although 12 fish were sampled per condition (four fish per tank), this data is presented using the number of tanks as replicates (*n* = 3) for proper determination of the bulk response.

### 2.3. Gene Expression Analyses

#### 2.3.1. RNA Extraction and cDNA Synthesis

Total RNA was extracted from ~100 mg of vertebral bone (*n* = 10) and white muscle (*n* = 8) tissues with 1 mL of TRI Reagent Solution (Applied Biosystems, Alcobendas, Spain) and homogenized on a Precellys^®^ Evolution homogenizer coupled to a Cryolys cooling unit (Bertin Instruments, Montigny-le-Bretonneux, France). Concentration and purity of the different samples were determined using a NanoDrop 2000 (Thermo Scientific, Alcobendas, Spain) and integrity was confirmed in a 1% agarose gel (*w*/*v*) stained with SYBR-Safe DNA Gel Stain (Life Technologies, Alcobendas, Spain). Next, 1100 ng of RNA were treated with DNase I (Life Technologies, Alcobendas, Spain) to remove all genomic DNA. Finally, RNA was retrotranscribed with the Transcriptor First Strand cDNA Synthesis Kit (Roche, Sant Cugat del Vallès, Spain) and the cDNA obtained stored at −20 °C for real-time quantitative PCR analyses (qPCR).

#### 2.3.2. Real-Time Quantitative PCR (qPCR)

The mRNA transcript levels of the target genes plus three reference genes for both tissues were examined in a CFX384^TM^ real-time system (Bio-Rad, El Prat de Llobregat, Spain). The primers used for each tissue are listed in Table S1. First, a dilution curve with a pool of samples was run to confirm primer efficiency, specificity of the reaction, absence of primer-dimers, and to determine the appropriate cDNA dilution for each assay. All the analyses were performed in triplicate wells using 384-well plates with 2.5 μL of iTaq Universal SYBR Green Supermix (Bio-Rad, El Prat de Llobregat, Spain), 250 nM of forward and reverse primers and 1 μL of diluted cDNA for each sample, in a final volume of 5 μL. All reactions were performed in the conditions previously described [26]. Moreover, three negative controls were also included and run in duplicate: no template control (NTC), no reverse transcriptase control (RTC), and PCR control (PCR, MilliQ water). The level of expression of each target gene was calculated relative to the geometric mean of the two most stable reference genes (ribosomal protein l27a (*rpl27a*) and ribosomal protein s18 (*rps18*) in bone; *rpl27a* and elongation factor 1 alpha (*ef1α*) in white muscle) using the ΔΔCq method [27]. Both reference genes stability and relative expression of the target genes were determined using the Bio-Rad CFX Manager Software v. 3.1 (Hercules, CA, USA).

### 2.4. Statistical Analyses

Data were analyzed using IBM SPSS Statistics v. 22 (IBM, Armonk, NY, USA) and are presented as the mean + SEM. As a biological replicate, the tank was used for growth parameters and the individual fish for gene expression results. Tank effect was checked on each parameter, but it was not observed. Identification of outliers and data normality and homoscedasticity were assessed by IQRs, Shapiro–Wilk and Levene’s test, respectively. Statistical significance between temperature groups was performed with Student’s *t*-test. One-way analysis of variance (one-way ANOVA) followed by Tukey’s post-hoc test was used for comparing among the three dietary groups at the same temperature. When homoscedasticity was not observed, Dunnett T3 test was applied. Statistical differences were considered significant for all analyses when *p* < 0.05. Data were plotted using GraphPad Prism v. 7 (GraphPad Software, La Jolla, CA, USA, www.graphpad.com).

## 3. Results

### 3.1. Somatic Growth Parameters in Response to Temperature and Diet

In fish fed with P diet, the temperature of 28 °C caused a significant increase in FBW, WG, SGR, and BL with respect to those reared at 21 °C. On the other hand, none of the parameters evaluated showed significant differences among the fish fed the three different experimental diets when maintained at 28 °C (Table 2). These results have been previously reported in Riera-Heredia et al. [22] but are also included in the present manuscript for a better discussion regarding growth regulation together with the newly generated gene expression data.

### 3.2. Effects of a High Rearing Temperature in Bone and White Skeletal Muscle

#### 3.2.1. GH-IGFs Axis Members and a Proliferation Marker

**Figure 2:**
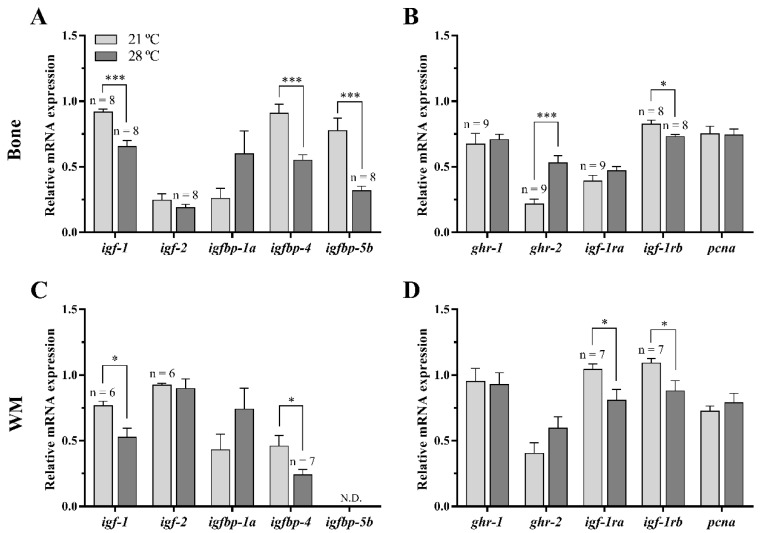
Relative gene expression of growth hormone-insulin-like growth factors (GH-IGFs) axis members and the proliferation marker *pcna* in (**A**,**B**) bone and (**C**,**D**) white skeletal muscle (WM) of gilthead sea bream juveniles fed with a palm oil (P) diet at 21 °C and 28 °C. Data are shown as the mean + SEM (*n* = 10 for bone and *n* = 8 for WM; if less, the *n* is indicated above each bar). Significant differences between temperature groups are indicated by asterisks (*p* < 0.05 shown as *; *p* < 0.001 ***).

In bone of fish fed with P diet, the high temperature caused a significant decrease in the gene expression of total *igf-1*, the IGF binding proteins *igfbp-4* and *igfbp-5b*, and the IGF-1 receptor *igf-1rb* compared to fish held at 21 °C. Contrarily, 28 °C significantly up-regulated the GH receptor *ghr-2* mRNA levels in fish bone compared to 21 °C-reared fish (Figure 2A,B). Regarding white muscle, the mRNA levels of *igf-1*, *igfbp-4*, *igf-1ra*, and *igf-1rb* were down-regulated at 28 °C, while *igfbp-5b* was not detectable in this tissue (Figure 2C,D). Besides, the mRNA levels of *igf-2*, *igfbp-1a* and *ghr-1* remained unaltered in both tissues, *igf-1ra* in bone and *ghr-2* in white muscle as well.

In addition, the expression of the commonly used proliferation marker, proliferating cell nuclear antigen (*pcna*), showed no differences between groups in any of the two tissues (Figure 2B,D).

#### 3.2.2. Osteogenic, Osteoclastic, and Myogenic Regulatory Factors

**Figure 3:**
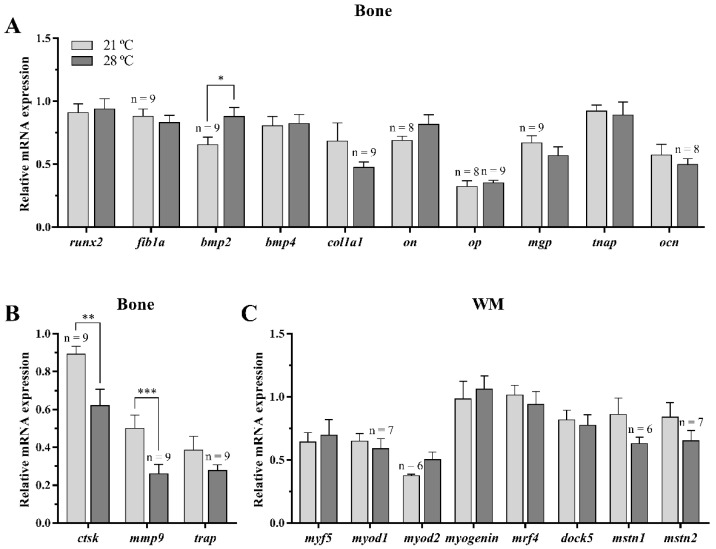
Relative expression of (**A**) osteogenic-, (**B**) osteoclastic-, and (**C**) myogenic-related genes in (**A**,**B**) bone and (**C**) white skeletal muscle (WM) of gilthead sea bream juveniles fed with a palm oil (P) diet at 21 °C and 28 °C. Data are shown as the mean + SEM (*n* = 10 for bone and *n* = 8 for WM; if less, the *n* is indicated above each bar). Significant differences between temperature groups are indicated by asterisks (*p* < 0.05 shown as *; *p* < 0.01 **; *p* < 0.001 ***).

Most of the osteogenesis-related genes determined in vertebra bone ((runt-related transcription factor 2 (*runx2*), fibronectin subunit 1a (*fib1a*), bone morphogenetic protein 4 (*bmp4*), collagen type 1 alpha-1 (*col1a1*), osteonectin (*on*), osteopontin (*op*), matrix gla protein (*mgp*), tissue non-specific alkaline phosphatase (*tnap*), and osteocalcin (*ocn*)) were unaffected by the increase in the water rearing temperature, except for *bmp2*, which was significantly increased in fish maintained at 28 °C (Figure 3A). With regards to the osteoclasts markers involved in bone matrix degradation, the gene expression levels of cathepsin K (*ctsk*) and matrix metalloproteinase 9 (*mmp9*) were significantly down-regulated in the 28 °C-held fish, although differences were not found in the case of tartrate-resistant acid phosphatase (*trap*) (Figure 3B).

Concerning the myogenic regulatory factors (*myf5*, *myod1*, *myod2*, *myogenin*, and *mrf4*), the myoblast fusion marker (*dock5*), as well as the negative regulators of muscle growth (the myostatins, *mstn1*, and *mstn2*), significant differences were not observed for any of them in response to the high temperature treatment under the experimental conditions tested (Figure 3C).

#### 3.2.3. Lipid Metabolism and Oxidative Stress-Related Genes

**Figure 4:**
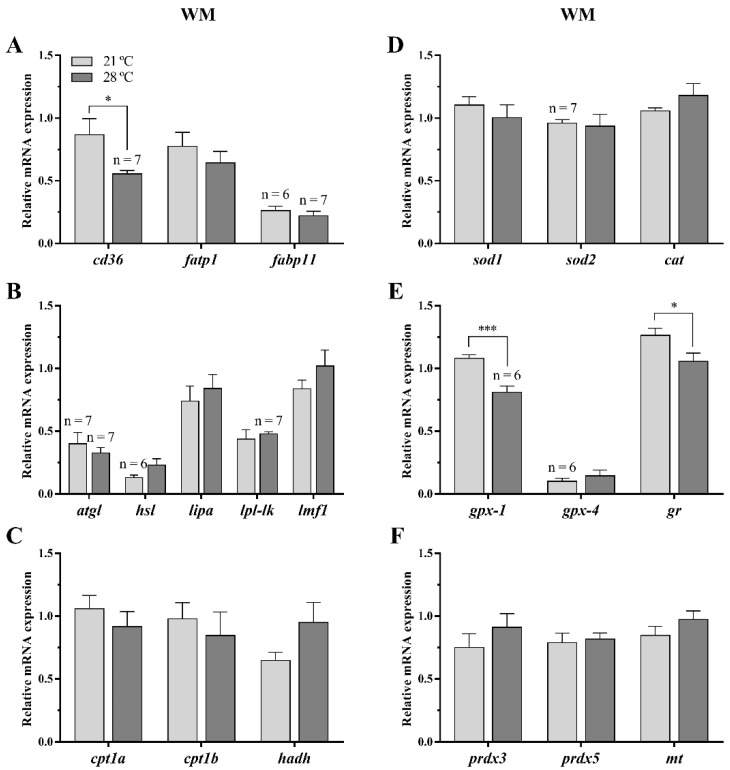
Relative gene expression of (**A**) fatty acid transporters, (**B**) lipases, (**C**) β-oxidation markers, (**D**) antioxidant factors, (**E**) glutathione metabolism-related, and (**F**) antioxidant enzymes in white skeletal muscle (WM) of gilthead sea bream juveniles fed with a palm oil (P) diet at 21 °C and 28 °C. Data are shown as the mean + SEM (*n* = 8; if less, the *n* is indicated above each bar). Significant differences between temperature groups are indicated by asterisks (*p* < 0.05 shown as *; *p* < 0.001 ***).

Lipid metabolism and oxidative stress-related genes were measured only in white skeletal muscle. The expression of most of the genes implicated in fatty acid transport ((fatty acid transport protein 1 (*fatp1*) and fatty acid binding protein 11 (*fabp11*)), lipid metabolism ((adipose triglyceride lipase (*atgl*), hormone sensitive lipase (*hsl*), lipase a (*lipa*), lipoprotein lipase-like (*lpl-lk*) and lipase maturation factor 1 (*lmf1*)) and *β*-oxidation ((carnitine palmitoyltransferase 1a (*cpt1a), cpt1b* and hydroxyacil-CoA dehydrogenase (*hadh*)) did not reveal any differences between temperature groups, except for the fatty acid translocase/cluster of differentiation (*cd36*), to which exposure to 28 °C significantly decreased its mRNA levels compared to those in fish reared at 21 °C (Figure 4A–C).

Concerning the oxidative stress markers, in fish fed with P diet, the temperature of 28 °C significantly reduced the gene expression of glutathione peroxidase-1 (*gpx-*1) and glutathione reductase (*gr*) compared to fish held at 21 °C. Differences were not observed for *gpx-4* and neither for the antioxidant factors ((superoxide dismutase 1 (*sod1*), *sod2* and catalase (*cat*)) and enzymes ((thioredoxin-dependent peroxide reductase 3 (*prdx3*), *prdx5* and metallothionein (*mt*)) analyzed (Figure 4D–F).

### 3.3. Effects of VO-Based Diets at a High Rearing Temperature in Bone and White Skeletal Muscle

**Figure 5:**
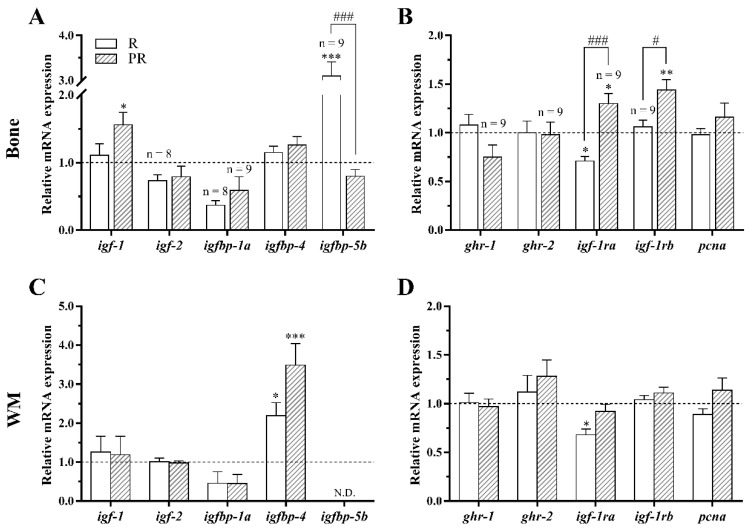
Relative expression of GH-IGFs axis-related members and the proliferation marker *pcna* in (**A**,**B**) bone and (**C**,**D**) white skeletal muscle (WM) of gilthead sea bream juveniles fed with the experimental diets containing rapeseed (R) or palm + rapeseed (PR) oils, expressed as fold change respect to those fish fed with a palm oil (P) diet (dotted line) and reared at 28 °C. Data are shown as the mean + SEM (*n* = 10 for bone and *n* = 8 for WM; if less, the *n* is indicated above each bar). Significant differences between fish fed with R or PR diets compared to those fed with P diet are indicated by asterisks and between animals fed R and PR diets with a hash (*p* < 0.05 shown as */#; *p* < 0.01 **; *p* < 0.001 ***/###).

**Figure 6:**
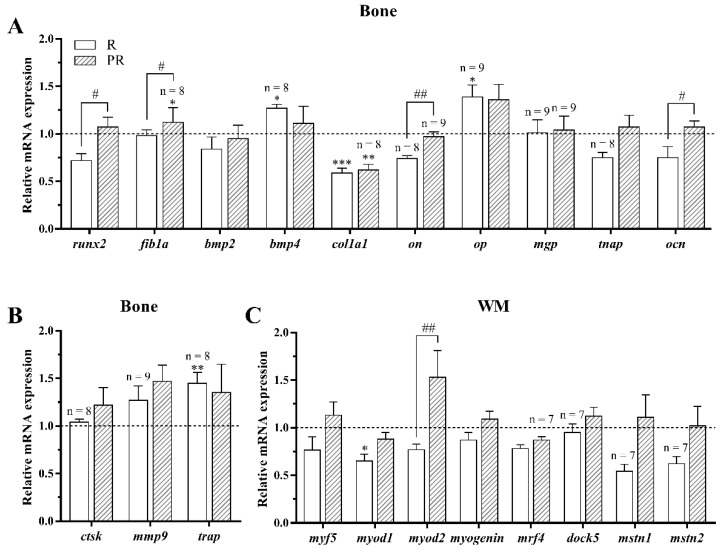
Relative expression of (**A**) osteogenic-, (**B**) osteoclastic-, and (**C**) myogenic-related genes in (**A**,**B**) bone and (**C**) white skeletal muscle (WM) of gilthead sea bream juveniles fed with the experimental diets containing rapeseed (R) or palm + rapeseed (PR) oils, expressed as fold change respect to those fish fed with a palm oil (P) diet (dotted line) and reared at 28 °C. Data are shown as the mean + SEM (*n* = 10 for bone and *n* = 8 for WM; if less, the *n* is indicated above each bar). Significant differences between fish fed with R or PR diets compared to those fed with P diet are indicated by asterisks and between animals fed R and PR diets with a hash (*p* < 0.05 shown as */#; *p* < 0.01 **/##; *p* < 0.001 ***).

**Figure 7:**
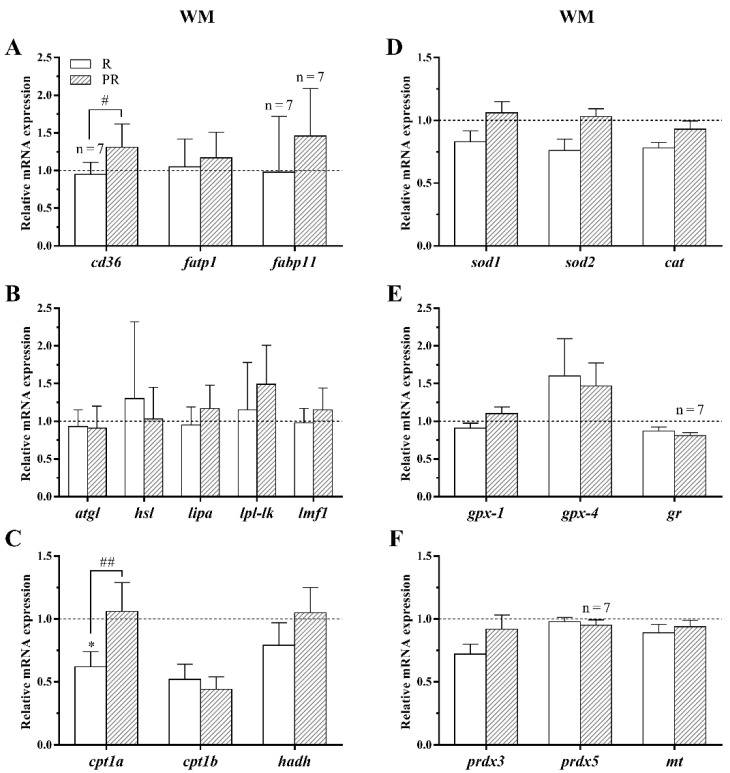
Relative gene expression of (**A**) fatty acid transporters, (**B**) lipases, (**C**) β-oxidation markers, (**D**) antioxidant factors, (**E**) glutathione metabolism-related, and (**F**) antioxidant enzymes in white skeletal muscle (WM) of gilthead sea bream juveniles fed with the experimental diets containing rapeseed (R) or palm + rapeseed (PR) oils, expressed as the fold change with respect to those fish fed with a palm oil (P) diet (dotted line) and reared at 28 °C. Data are shown as the mean + SEM (*n* = 8; if less, the *n* is indicated above each bar). Significant differences between fish fed with R or PR diets compared to those fed with P diet are indicated by asterisks and between animals fed R and PR diets with a hash (*p* < 0.05 shown as */#; *p* < 0.01 ##).

The same clusters of genes and tissues were analyzed in the fish fed the R and PR diets at 28 °C, and results are expressed as the fold change with respect to those from the fish fed with P diet (dotted line) at 28 °C (Figure 5, Figure 6 and Figure 7).

#### 3.3.1. GH-IGFs Axis Members and a Proliferation Marker

In bone from juveniles fed with PR diet, the gene expression of total *igf-1* significantly increased when comparing to fish fed P diet, and the receptors (*igf-1ra* and *igf-1rb*) were also significantly up-regulated with respect to both, the P and R groups. On the other hand, *igfbp-5b* was significantly enhanced in the bone of fish fed with R diet compared to fish fed P and PR diets. Finally, *igf-2*, *igfbp-1a*, *igfbp-4*, *ghr-1*, *ghr-2*, and *pcna* expression did not show significant differences among groups in this tissue (Figure 5A,B).

The analysis of GH-IGFs axis-related genes expression in the white muscle of fish maintained at 28 °C revealed that differences did not exist among dietary groups for most of them, but *igfbp-4* was significantly up-regulated in fish fed both R and PR diets compared to the P group. Furthermore, *igf-1ra* mRNA levels were significantly lower in R diet-fed animals than in those fed with P diet. The other members of the axis and *pcna* remained in this tissue unaltered among groups (Figure 5C,D).

#### 3.3.2. Osteogenic, Osteoclastic and Myogenic Regulatory Factors

In bone tissue, PR diet significantly increased the mRNA levels of *runx2*, *fib1a*, *on*, and *ocn* compared to R diet and the expression of *fib1a* also in comparison to P diet. In addition, *bmp4* and *op* gene expression was significantly enhanced in fish fed with R diet compared to P group, while both fish fed the R and PR diets had decreased mRNA levels of *col1a1* respect to P diet-fed juveniles (Figure 6A). On the other hand, *trap* was the only osteoclast marker affected by the dietary intervention at high temperature, with its gene expression being significantly higher in animals fed with R diet than in those fed with P diet, whereas *ctsk* and *mmp9* remained unaltered (Figure 6B).

In white muscle from gilthead sea bream fed with R diet, significantly decreased transcript levels of *myod1* with respect to P diet-fed fish were observed, while *myod2* was significantly up-regulated in fish fed with PR diet when compared with the R group. The remaining genes were not altered among the three VO-based diet groups reared at 28 °C (Figure 6C).

#### 3.3.3. Lipid Metabolism and Oxidative Stress-Related Genes

Transcript levels of *cd36* were found to significantly increase in white muscle from fish fed with the PR diet in comparison with R diet-fed fish. Besides, a down-regulation of *cpt1a* mRNA levels was detected in animals fed with R diet compared to the other two conditions. The remaining genes analyzed were not affected among the three dietary groups evaluated at 28 °C (Figure 7A–C).

Finally, differences were not found in the transcript levels of any of the oxidative stress-related genes in response to VO diets at a high rearing temperature in the white muscle of this species (Figure 7D–F).

## 4. Discussion

Although the use of different VO in fish feeds formulation has been widely studied in the last decades, many aspects remain still unexplored, such as its physiological effects on bone tissue growth, or the possible combined impacts of dietary VO inclusion with a high-water rearing temperature. The present study analyzed somatic growth and the transcriptional regulation of the musculoskeletal endocrine system and endogenous growth modulators, as well as white muscle lipid metabolism and oxidative stress-related genes in gilthead sea bream juveniles fed with three partially substituted FO diets by two different VO (i.e., PO, RO, or the combination of both oils) in an increased water temperature context.

The growth of vertebrates, including fish, is primarily mediated through the endocrine GH-IGFs axis. In fact, the actions of GH and IGFs occur via binding to their corresponding receptors in target tissues, while at the same time, IGF-1 function can be influenced by different types of binding proteins [28]. In the current study, the high rearing temperature of 28 °C significantly increased the somatic growth of gilthead sea bream fed with P diet, in comparison to the fish reared at 21 °C, demonstrated by the higher FBW, WG, SGR, and BL values observed. Parallel to this, in the same animals, a temperature of 28 °C resulted in a general decrease of the gene expression of members of the GH-IGFs axis identified as growth promoting indicators (including *igf-1*, the binding proteins *igfbp-4*, *igfbp-5b* and *igf-1rs*) in white muscle and/or bone, suggesting the existence of negative feedback mechanisms to adjust the expression of these factors when conditions for growth are favorable to avoid uncoupled overgrowth. On the other hand, *igf-2* and *gh receptors* (*ghr-1* and *ghr-2*) remained unaltered or increased in the case of *ghr-2* in bone, supporting the recognized major involvement of IGF-1, compared to the other components of the hormonal axis such as GH, directly controlling musculoskeletal growth in fish [29,30,31]. This GH-IGFs axis gene expression pattern is in agreement with that previously described in a shorter trial (i.e., 3 days) with the same species reared as well at 28 °C temperature [26]. In addition, the up-regulating effect of high temperature on *ghr-2* observed in this study in bone was also reported in the liver of rainbow trout (*Oncorhynchus mykiss*) juveniles [32] and embryos of the same species [33]. In fact, it is known that the two isoforms of the GH receptor are differentially regulated depending on the tissue, in response to a temperature change [32], which agrees with the results observed in the present work. Furthermore, an increase (not significant) of *igfbp-1a*, an important growth and development inhibitor [34], was also observed in both tissues in agreement with Balbuena-Pecino et al. [26]. All in all, the transcriptional modulation of GH-IGFs axis members in gilthead sea bream caused by the elevation of water rearing temperature to 28 °C appears to be contributing to a critical turnover regulation of the musculoskeletal system to find growth homeostasis.

Concerning muscle and bone specific local regulators, very little differences were observed in the present study in response to a high rearing temperature, with the most remarkable changes being the down-regulation of the bone resorption genes *ctsk* and *mmp9*, while the osteogenesis-related genes remained unaltered except for *bmp2*, which was significantly up-regulated. Like in mammals, resorption and remodeling of teleost fish bone tissue are essential processes for development and growth [35], in part controlled by IGF-1, known to stimulate both osteoblast proliferation as well as osteoclast differentiation and activity [36,37]. In this context, the reduction in the mRNA levels of *ctsk* and *mmp9* could be the result of a regulatory mechanism involving IGF-1 favoring bone formation [35]. On the other hand, several studies have demonstrated that BMP2-mediated signals not only induce osteoblasts but are also involved in activating bone resorption [38,39,40], thus balancing the effects commented. Therefore, we can speculate that bone turnover is only mildly influenced by temperature, as previously shown in other vertebrate species such as dairy cows [41], but overall, has an apparent net effect towards bone matrix formation rather than resorption.

As existing literature reports, increasing water temperature has been demonstrated to coincide with higher growth in different fish species including gilthead sea bream [42,43,44,45,46]. In fact, for most species, a slight increase in their normal range of temperature is known to be beneficial as it results in more energy intake, higher rates of diffusion and enzyme-substrate complexes and, as a consequence, higher reaction rates for growth [47]. Nevertheless, if this rise is excessive, it can induce an unfavorable condition for balanced musculoskeletal growth [48]. According to this and summarizing, the present results suggest that, in gilthead sea bream, the GH-IGFs axis is locally modulated by high temperature in bone and white muscle, in an attempt to counteract the accelerated growth rate of these animals in an elevated water temperature context as a compensatory response. Furthermore, white muscle and particularly bone, reducing the expression of osteoclastogenic genes, respond to support harmonic musculoskeletal growth upon high temperature, which could prevent in the long-term the appearance of skeletal anomalies.

Regarding feeding the various experimental diets P, R, or PR in fish maintained at 28 °C, differences in growth were not found, indicating that the three diets appear to be adequate for gilthead sea bream to grow at this temperature. However, PR diet increased the mRNA levels of *igf-1* and both *igf-1rs* in bone, and *igfbp-4* in white muscle suggesting an induction of growth enhancement, as generally these parameters are associated with high growth rates, thus readjusting the down-regulating effects over the GH-IGFs axis caused by elevated temperature. In a previous study, in gilthead sea bream fed with diets formulated with graded levels of a VO mixture (RO:LO:PO) replacing FO at 33%, 66%, and 100%, a decreased growth of the fish fed with the VO diets was found together with reduced plasma levels of IGF-1 and mRNA levels of *igf-1* and *ghr-1* [19]. Regarding substituted diets by individual VO (up to total FO replacement), several studies have reported either no changes or less growth in comparison to a FO diet [10,13,49,50,51,52]. Nevertheless, Sánchez-Moya et al. [14] recently described increased growth in gilthead sea bream fed diets with high PO content after an 18-week trial, although in that study a diet containing only FO was not evaluated. In this context, differences among studies can be explained by several parameters such as the type and percentage of VO inclusion and the duration of the experimental trial.

In our previous study, from which this is a continuation, the liver and adipose tissue data obtained indicated that the PR diet could be for gilthead sea bream the most equilibrated to grow at a high rearing temperature while avoiding excessive fat accumulation [22]. Supporting this, and concerning the current transcriptional analysis of osteogenic and myogenic markers, the fish fed with the PR diet also have, in comparison to the fish fed the other two diets, increased expression of early (*runx2* and *fib1a*) and late (*on* and *ocn*) pro-osteoblastic genes in bone [53] and of an inducer of myoblast proliferation *myod2* in muscle [54]. Therefore, this combination of PO and RO in the feed appears once more to be optimal for gilthead sea bream production to obtain good growth rates with potentially reduced skeletal anomalies, especially under elevated temperature conditions (i.e., high energy requirement).

In the present study, most of the lipid metabolism-related genes analyzed in muscle were not affected by temperature, but *cd36* expression was significantly reduced in fish reared at 28 °C compared with the 21 °C-group, suggesting decreased fatty acid uptake in this tissue. Moreover, the down-regulated expression of this gene was also observed in adipose tissue and liver, and *fabp11* as well in the latter, in the same experimental animals as previously reported [22]. Contrary to this, an increase in the muscle gene expression of *cd36* and *fabp11* was observed in response to high water temperature after only a three days exposure [26]. Moreover, elevated mRNA levels of the lipases *lipa* and *lpl-lk* as well as of the mitochondrial uncoupling protein 2 (*ucp2*) were also found in the short-term study, suggesting increased use of fatty acids, although in a less efficient manner, as a first response to the new environmental conditions. However, in the current long-term dietary trial, we can hypothesize that most differences between fish from the two temperature groups were not significant, as the fish had already adapted to the more energy demanding situation.

Differences in expression were neither observed concerning the lipid metabolism-related genes nor when feeding the fish with the two other VO-based diets at 28 °C. Nevertheless, fish fed with the PR diet showed an upregulated expression of *cd36* and *cpt1a* compared to those fish fed with R diet, suggesting an increase in fatty acid uptake as well as oxidation in the former animals, since the flux of *β*-oxidation is primarily determined by CPT1 [55]. This is in agreement with the upregulated *cpt-1a* gene expression observed in the muscle of gilthead sea bream fed blended VO diets [14] and in the increase in lipid turnover in terms of key genes expression in the liver of the same fish fed PR diet [22]. Likewise, FO replacement by LO and SO also induced increased activities of the muscle β-oxidation enzymes CPT1 and 3-hydroxyacyl-CoA dehydrogenase (HOAD) in gilthead sea bream, independently of its inclusion level in the diet [56]. Moreover, Ofori-Mensah et al. [57] has shown in the same species that the gene expression of *cpt1a* and *cpt1b*, as well as of the peroxisome proliferator-activated receptors *pparα* and *pparβ*, increased in juveniles fed diets containing VO (camelina or chia oils), indicating enhanced fatty acid oxidation. In Atlantic salmon (*Salmo salar*), white muscle exhibited also an increased *β*-oxidation capacity when the dietary RO content was raised from 25 to 75%, indicating a positive correlation between these two parameters [58]. However, differences were not observed when feeding the same species with several VO diets containing PO, capelin oil, oleic acid-enriched sunflower oil, or a mixture of sunflower oil and capelin oil [59]. In fact, changes in dietary fatty acid composition are known to be able to directly influence fatty acid catabolism, lipid transport and uptake, or lipogenesis and the lipid composition of the flesh, being the effects clearly associated to the specific combination and level of VO and the species analyzed [60]. Indeed, dietary VO composition has only moderately affected muscle lipid metabolism in the gilthead sea bream of the present study, at least from the point of view of transcriptional changes.

Finally, concerning oxidative stress, which is the disturbance in the prooxidant-antioxidant balance in favor of the former [61], a general lack of response was found in the present study for most of the genes analyzed according to the temperature and dietary treatments. However, in fish fed with P diet, the temperature of 28 °C reduced the mRNA levels of two enzymes associated with the antioxidant glutathione (GSH) metabolism (*gpx-1* and *gr*) compared to those in the 21 °C-held fish. Recent seasonal studies in gilthead sea bream have shown that oxidative stress seems to be more prominent during warming because of the increased aerobic metabolism [62]. In a previous study in goldfish (*Carassius auratus*), GSH-dependent system, but no other antioxidant enzymes such as SOD or CAT, showed the most marked response in a transition from low to high temperature [63], being the first enzymatic lines of defense. GPx contributes to protect from oxidative stress-induced damage decreasing soluble hydroperoxides, at the expenses of GSH. The antioxidant capacity of this molecule relies on the ability of GSSG (the oxidized form) to be readily reduced back to GSH by GR [64,65]. In this context, high GSSG levels could indicate a situation of enhanced oxidative stress, while decreased GSH levels have been specifically associated with increased lipid peroxidation [66,67], which has been related to negatively impact on the flavor, color and nutritional characteristics of the flesh in fish [68]. Thus, in our model, the down-regulation of *gpx-1* and *gr* in white muscle could indicate a lower protective role by GSH system against oxidative damage and negative reactive oxygen species (ROS) effects in this tissue under a high temperature situation. Nevertheless, we need to have in mind that changes in transcript levels do not always coincide with protein levels and/or enzyme activities. In fact, in skeletal muscle of striped bass (*Morone saxatilis*), significant changes were not found after six weeks at a warm temperature in the protein levels of GPx1, GPx4, and glutathione-S-transferase (GST) and neither in the enzymatic activities of GPx and GST [69]. Other studies have also reported an association between increasing temperatures and oxidative stress, although these effects seem to be both tissue and species specific. Levels of lipid peroxidation and CAT activity in European sea bass (*Dicentrarchus labrax*) and of lipid peroxides and thiobarbituric-acid reactive substances (TBARs) in goldfish increased with temperature but returned to control values with time or after temperature recovery [70,71]. Thus, it cannot be excluded that, in our trial, a shorter sampling time could have also revealed the increased expression of these genes as an initial response to thermal stress, but instead, the general lack of response observed could point out that, after two months, fish have already acclimatized to the elevated temperature.

In line with this and considering dietary treatments, differences were not observed in the present study concerning the oxidative stress-related genes expression in the gilthead sea bream reared at 28 °C and fed with either one of the three VO diets. Indeed, although skeletal muscle was not specifically analyzed in most studies, in a longer feeding trial (13 weeks) with the same species, replacing FO by a blend of VO reduced liver lipid peroxidation while enhancing GSH redox status, GPx, and GR enzymatic activities, improving fish oxidative status [72]. Likewise, total FO substitution by different VO including PO or RO, also decreased hepatic anti-oxidant capacity in tilapia (*Oreochromis niloticus*) [51] and large yellow croaker (*Larimichthys crocea*) [73]. The same pro-oxidant effect was found through diminishing mRNA values of some of the antioxidant-related genes (e.g., *cat*, *sod*, *gst*, *gr*, *gpx*) in rainbow trout fed a 100% LO-based diet [74]. Overall, these data confirm the importance of fine-tuning the amount of VO on diets, as its effects on oxidative stress vary accordingly.

## 5. Conclusions

To sum up, the present study reports that in gilthead sea bream juveniles fed with a diet rich in PO, the GH-IGFs axis is transcriptionally modulated by elevated water temperature (28 °C) in both bone and white muscle tissues by a possible negative feedback mechanism to balance the higher growth context and to avoid the uncoupled overgrowth of these animals. On the other hand, temperature did not affect most of the lipid metabolism and oxidative stress markers analyzed in white muscle, reflecting an achieved adapted situation, although showing that this tissue has a lower protective role of the GSH system. In addition, from the three dietary treatments evaluated, the PR diet seems to be for gilthead sea bream the most beneficial one in terms of promoting an optimum endocrine environment for balanced musculoskeletal growth at high water temperature conditions while enhancing muscular fatty acid uptake and oxidation. Furthermore, the dietary use of either PO, RO, or the combination of both oils with up to 65% of FO replacement seems not to alter antioxidant defenses. As a final point, these results demonstrate the importance, for proper growth and muscle metabolic and oxidative status, of taking into account the predicted global world temperature increments, when evaluating the substitution of raw materials by plant-based alternative ingredients in new feeds for this species.

## Figures and Tables

**Table 1:** Composition of the experimental diets.

**Ingredients (%)**	**P**	**R**	**PR**
Wheat	6.83	6.83	6.83
Corn meal	10	10	10
Wheat gluten	14.79	14.78	14.78
Soya protein	25	25	25
Broad beans	10	10	10
Fishmeal	20	20	20
Fish oil	5.34	5.34	4.45
Linseed oil	1.33	2	1.33
Rapeseed oil	0	6.67	3.34
Palm oil	6.67	0	3.55
Phosphate	0.03	0.03	0.03
**Composition (%)**			
Moisture	7.6	7.7	7.5
Protein	51.9	51.8	51.7
Fat	19.2	18.9	19.4
Ash	5.7	5.7	5.6

P: palm; R: rapeseed; PR: palm + rapeseed.

**Table 2:** Growth parameters and somatic indexes of fish fed with the experimental diets P at 21 °C or P, R, and PR at 28 °C for 2 months.

	21 °C	28 °C		
Biometrics	P	P	R	PR
IBW (g)	24.21 ± 0.57	22.89 ± 0.40	22.80 ± 0.75	22.24 ± 0.77
FBW (g)	54.32 ± 1.78 *	68.02 ± 2.34	63.33 ± 2.09	62.21 ± 3.80
WG (%)	124.3 ± 5.19 *	197.5 ± 13.27	177.8 ± 2.33	179.2 ± 7.62
SGR (%)	1.52 ± 0.04 *	2.05 ± 0.08	1.93 ± 0.02	1.94 ± 0.05
BL (cm)	15.06 ± 0.02 *	16.55 ± 0.11	16.37 ± 0.07	16.23 ± 0.22
CF (%)	1.59 ± 0.06	1.5 ± 0.04	1.44 ± 0.03	1.45 ± 0.03

Data are shown as the mean ± SEM (*n* = 3 tanks). Asterisks (*) indicate significant differences between fish fed with P diet at different temperatures. No differences were observed among fish fed the different diets at high temperature (*p* < 0.05). Initial body weight (IBW); final body weight (FBW); weight gain (WG) [(FBW − IBW/IBW) × 100]; somatic growth rate (SGR) [((ln FBW − ln IBW)/time) × 100], where time was 53 days; body length (BL); condition factor (CF) [(FBW/BL3) × 100]. P: palm; R: rapeseed; PR: palm + rapeseed.

## Data Availability

The data presented in this study are available in the current article and its corresponding supplementary material.

## Supplementary Materials

The following are available online at https://www.mdpi.com/2076-2615/11/2/260/s1, Table S1: Primers used in the Real-Time quantitative PCR analyses.
