# Supplementary material for: Musculoskeletal Growth Modulation in Gilthead Sea Bream Juveniles Reared at High Water Temperature and Fed with Palm and Rapeseed Oils-Based Diets
Source: Animals (Basel). 2021 Jan 21;11(2):260. doi: 10.3390/ani11020260 (PMC7909841; doi:10.3390/ani11020260)
Supplement: Supplementary file 1 [file animals-11-00260-s001.pdf]

Table S1. Primers used in the Real-Time quantitative PCR analyses

Type	Gene	Primer Sequences (5′–3′)	Ta (ºC)	Accession Nnumber
Reference	*rpl27a*	**F**: AAGAGGAACACAACTCACTGCCCCAC<br>**R**: GCTTGCCTTTGCCCAGAACTTTGTAG	68	AY188520
	rps18	**F**: GGGTGTTGGCAGACGTTAC<br>**R**: CTTCTGCCTGTTGAGGAACCA	60	AM490061.1
	ef1α	**F**: CTTCAACGCTCAGGTCATCAT<br>**R**: GCACAGCGAAACGACCAAGGGGA	60	AF184170
GH-IGFs axis	*igf-1*	**F**: GAGACCTAGTGGAGGCTGTC<br>**R**: TCTTGTTGATGGTGAGCTGT	60	EF688016
	igf-2	**F**: TGGGATCGTAGAGGAGTGTTGT<br>**R**: CTGTAGAGAGGTGGCCGACA	60	AY996778
	igfbp-1a	**F**: AGTGCGAGTCCTCTCTGGAT<br>**R**: TCTCTTTAAGGGCACTCGGC	60	KM522771
	igfbp-4	**F**: TCCACAAACCAGAGAAGCAA<br>**R**: GGGTATGGGGATTGTGAAGA	68	F5T95CD02JMZ9K
	igfbp-5b	**F**: TTTCTCTCTCGGTGTGC<br>**R**: TCAAGTATCGGCTCCAG	60	AM963285
	ghr-1	**F**: ACCTGTCAGCCACCACATGA<br>**R**: TCGTGCAGATCTGGGTCGTA	60	AF438176
	ghr-2	**F**: GAGTGAACCCGGCCTGACAG<br>**R**: GCGGTGGTATCTGATTCATGGT	60	AY573601
	igf-1ra	**F**: AGCATCAAAGACGAACTGG<br>**R**: CTCCTCGCTGTAGAAGAAGC	55	KT156846
	igf-1rb	**F**: GCTAATGCGAATGTGTTGG<br>**R**: CGTCCTTTATGCTGCTGATG	55	KT156847
Proliferation	*pcna*	**F**: TGTTTGAGGCACGTCTGGTT<br>**R**: TGGCTAGGTTTCTGTCGC	58	NM_131404.2
Osteogenesis	*runx2*	**F**: ACCCGTCCTACCTGAGTCC<br>**R**: AGAAGAACCTGGCAATCGTC	60	JX232063
	fib1a	**F**: CGGTAATAACTACAGAATCGGTGAG<br>**R**: CGCATTTGAACTCGCCCTTG	60	FG262933
	bmp2	**F**: GGAGAAGCAGCGTGGATTAAACACGAAT<br>**R**: GGCCTGCGCCTCAGTCCAAACATATT	65	AY500244
	bmp4	**F**: CACGCCATTGTTCAGACACT<br>**R**: GCCCTCCACTACCATTTCCT	60	FJ436409
	col1a1	**F**: GAGATGGCGGTGATGTGGCGGAGTC<br>**R**: GCCTGGTTTGGCTGGATGAAGAGGG	68	DQ324363
	on	**F**: AGGAGGAGGTCATCGTGGAAGAGCC<br>**R**: GTGGTGGTTCAGGCAGGGATTCTCA	68	AY239014
	op	**F**: AAAACCCAGGAGATAAACTCAAGACAACCCA<br>**R**: AGAACCGTGGCAAAGAGCAGAACGAA	68	AY651247

	mgp	**F**: TGTGTAATTTATGTAGTTGTTCTGTGGCATCTCC<br>**R**: CGGGCGGATAGTGTGAAAAATGGTTAGTG	68	AY065652
	tnap	**F**: CATCGCAACCCTTTTCACAGTCACCCG<br>**R**: AACAGTGCCCAAACAGTGGTCCCATTAGC	68	AY266359
	ocn	**F**: TCCGCAGTGGTGAGACAGAAG<br>**R**: CGGTCCGTAGTAGGCCGTGTAG	60	AF048703
**Osteoclastic**	*ctsk*	**F**: AGCGAGCAGAACCTGGTGGAC<br>**R**: GCAGAGTTGTAGTTGGGGTCGTAG	60	DQ875329
	mmp9	**F**: ATTCAGAAGGTGGAGGGAGCG<br>**R**: CATTGGGGACACCACCGAAGA	60	AM905938
	trap	**F**: CTTAATCGTTGCCATCCCTGTG<br>**R**: CTCCCATCTGCTCTGCTACTTTG	60	FM147928
**Myogenesis**	*myf5*	**F**: CTACGAGAGCAGGTGGAGAACT<br>**R**: TGTCTTATCGCCCAAAGTGTC	64	JN034420
	myod1	**F**: TTTGAGGACCTGGACCC<br>**R**: CTTCTGCGTGGTGATGGA	60	AF478568.1
	myod2	**F**: CACTACAGCGGGGATTCAGAC<br>**R**: CGTTTGCTTCTCCTGGACTC	60	AF478569
	myogenin	**F**: CAGAGGCTGCCCAAGGTGGAG<br>**R**: CAGGTGCTGCCCGAACTGGGCTCG	68	EF462191
	mrf4	**F**: CATCCCACAGCTTTAAAGGCA<br>**R**: GAGGACGCCGAAGATTCACT	60	JN034421
	dock5	**F**: TCAACAGGCCCAGTAAATCC<br>**R**: GGGAAGCAGTTCCATCATTC	60	SRR278741 isotig14100
**Myostatins**	*mstn1*	**F**: GTACGACGTGCTGGGAGACG<br>**R**: CGTACGATTCGATTCGCTTG	60	AF258448.1
	mstn2	**F**: ACCTGGTGAACAAAGCCAAC<br>**R**: TGCGGTTGAAGTAGAGCATG	60	AY046314
**FA transporters**	*cd36*	**F**: GTCGTGGCTCAAGTCTTCCA<br>**R**: TTTCCCGTGGCCTGTATTCC	60	ERR12611_isotig20793
	fatp1	**F**: CAACAGAGGTGGAGGGCATT<br>**R**: GGGGAGATACGCAGGAACAC	60	ERR12611_isotig43042
	fabp11	**F**: CATTTGAGGAGACCACCGCT<br>**R**: ACTTGAGTTTGGTGGTACGCT	60	ERR12611_isotig32312
**Lipases**	*atgl*	**F**: GTGCTTCAGTCCTGGATGTCTTC<br>**R**: AGCCTTGCAGGTCCATGTTGA	60	JX975711
	hsl	**F**: GCTTTGCTTCAGTTTACCACCATTTC<br>**R**: GATGTAGCGACCCTTCTGGATGATGTG	60	EU254478
	lipa	**F**: TACTACATCGGACACTCTCAAGGAAC<br>**R**: GTGGAGAACGCTATGAATGCTATCG	60	JQ308831
	lpl-lk	**F**: CAGAGATGGAGCCGTCACTCAC<br>**R**: TCTGTCACCAGCAGGAACGAATG	60	JQ390609
	lmf1	**F**: CGGCTGGACTGGCTCATGT	60	JX975718

		**R**: CTCACTCTGCTCGTAGGTCTGGAA		
**β-oxidation**	*cpt1a*	**F**: GTGCCTTCGTTCGTTCCATGATC<br>**R**: TGATGCTTTATCTGCTGCCTGTTTG	60	JQ308822
	cpt1b	**F**: CCACCAGCCAGACTCCACAG<br>**R**: CACCACCAGCACCCACATATTTAG	60	DQ866821
	hadh	**F**: GAACCTCAGCAACAAGCCAAGAG<br>**R**: CTAAGAGGCGGTTGACAATGAATCC	60	JQ308829
**Oxidative stress**	*sod1*	**F**: CCATGGTAAGAATCATGGCGG<br>**R**: CGTGGATCACCATGGTTCTG	60	-
	sod2	**F**: CCTGACCTGACCTACGACTATGG<br>**R**: AGTGCCTCCTGATATTTCTCCTCTG	60	J0308833
	cat	**F**: TTCCCGTCCTTCATTCACTC<br>**R**: CTCCAGAAGTCCCACACCAT	60	JQ308823
	gpx-1	**F**: GAAGGTGGATGTGAATGGAAAAGATG<br>**R**: CTGACGGGACTCCAAATGATGG	63	DQ524992
	gpx-4	**F**: TGCGTCTGATAGGGTCCACTGTC<br>**R**: GTCTGCCAGTCCTCTGTCGG	61	AM977818
	gr	**F**: CAAAGCGCAGTGTGATTGTGG<br>**R**: CCACTCCGGAGTTTTGCATTTC	60	AJ937873
	prdx3	**F**: ATCAACACCCCACGCAAGACTG<br>**R**: ACCGTTTGGATCAATGAGGAACAGACC	65.5	GQ252681
	prdx5	**F**: GAGCACGGAACAGATGGCAAGG<br>**R**: TCCACATTGATCTTCTTCACGACTCC	64.5	GQ252683
	mt	**F**: CTCTAAGACTGGAACCTG<br>**R**: GGGCAGCATGAGCAGCAG	54	-

F: forward; R: reverse; Ta: annealing temperature; *rpl27a*: ribosomal protein l27a; *rps18*: ribosomal protein s18; *ef1α*: elongation factor 1 alpha; *igf-1*: insulin-like growth factor 1; *igf-2*: insulin-like growth factor 2; *igfbp-1a*: insulin-like growth factor binding protein 1a; *igfbp-4*: insulin-like growth factor binding protein 4; *igfbp-5b*: insulin-like growth factor binding protein 5b; *ghr-1*: growth hormone receptor 1; *ghr-2*: growth hormone receptor 2; *igf-1ra*: insulin-like growth factor 1 receptor a; *igf-1rb*: insulin-like growth factor 1 receptor b; *pcna*: proliferating cell nuclear antigen; *runx2*: runt-related transcription factor 2; *fib1a*: fibronectin subunit 1a; *bmp2*: bone morphogenetic protein 2; *bmp4*: bone morphogenetic protein 4; *col1a1*: collagen type 1 alpha-1; *on*: osteonectin; *op*: osteopontin; *mgp*: matrix gla protein; *tnap*: tissue non-specific alkaline phosphatase; *ocn*: osteocalcin; *ctsk*: cathepsin k; *mmp9*: matrix metalloproteinase 9; *trap*: tartrate-resistant acid phosphatase; *myf5*: myogenic factor 5; *myod1*: myogenic differentiation 1; *myod2*: myogenic differentiation 2; *myogenin*: myogenin; *mrf4*: myogenic regulatory factor 4; *dock5*: dedicator of cytokinesis 5; *mstn1*: myostatin 1; *mstn2*: myostatin 2; *cd36*: cluster of differentiation 36; *fatp1*: fatty acid transport protein 1; *fabp11*: fatty acid binding protein 11; *atgl*: adipose triglyceride lipase; *hsl*: hormone sensitive lipase; *lipa*: lipase a; *lpl-lk*: lipoprotein lipase-like; *lmf1*: lipase maturation factor 1; *cpt1a*: carnitine palmitoyltransferase 1a; *cpt1b*: carnitine palmitoyltransferase 1b; *hadh*: hydroxyacil-CoA dehydrogenase; *sod1*: superoxide dismutase 1; *sod2*: superoxide dismutase 2; *cat*: catalase; *gpx-1*: glutathione peroxidase 1; *gpx-4*: glutathione peroxidase 4; gr: glutathione reductase; prdx3: thioredoxin-dependent peroxide reductase 3 ; prdx5: thioredoxin-dependent peroxide reductase 5; mt: metallothionein.
